# Fragmented central venous catheter retained for years with minimal symptoms

**DOI:** 10.1002/ccr3.1041

**Published:** 2017-06-15

**Authors:** Clancy W. Mullan, Umair Tariq, Mark Goldin

**Affiliations:** ^1^ Hofstra Northwell School of Medicine Hofstra University Hempstead New York; ^2^ Department of Medicine Hofstra Northwell School of Medicine Long Island Jewish Medical Center New Hyde Park New York

**Keywords:** Central venous catheter, chest pain, fracture, interventional radiology, total parenteral nutrition

## Abstract

Unidentified fractures of indwelling tunneled catheters are presumably rare and possibly underreported, but their consideration is important in patients with a history of catheter placement with otherwise unexplained, atypical chest pain.

## Case Description

A 29‐year‐old woman with drug‐refractory hyperemesis gravidarum requiring total parental nutrition via indwelling tunneled catheter 6 years prior to admission presented with a prolonged history of intermittent chest pain. The pain was 5/10 in severity, present on deep inspiration or with large meals, was attributed to gastroesophageal reflux disease refractory to medication and lifestyle modification, and typically resolved within minutes. The patient had no other symptoms or relevant history, attributing the pain to scarring from catheter placement. The patient presented to the emergency department after visiting a new primary care provider for the first time, who performed a chest X‐ray in the office and, after noticing the fragment in the context of her intermittent pain, recommended emergency care.

**Figure 1:**
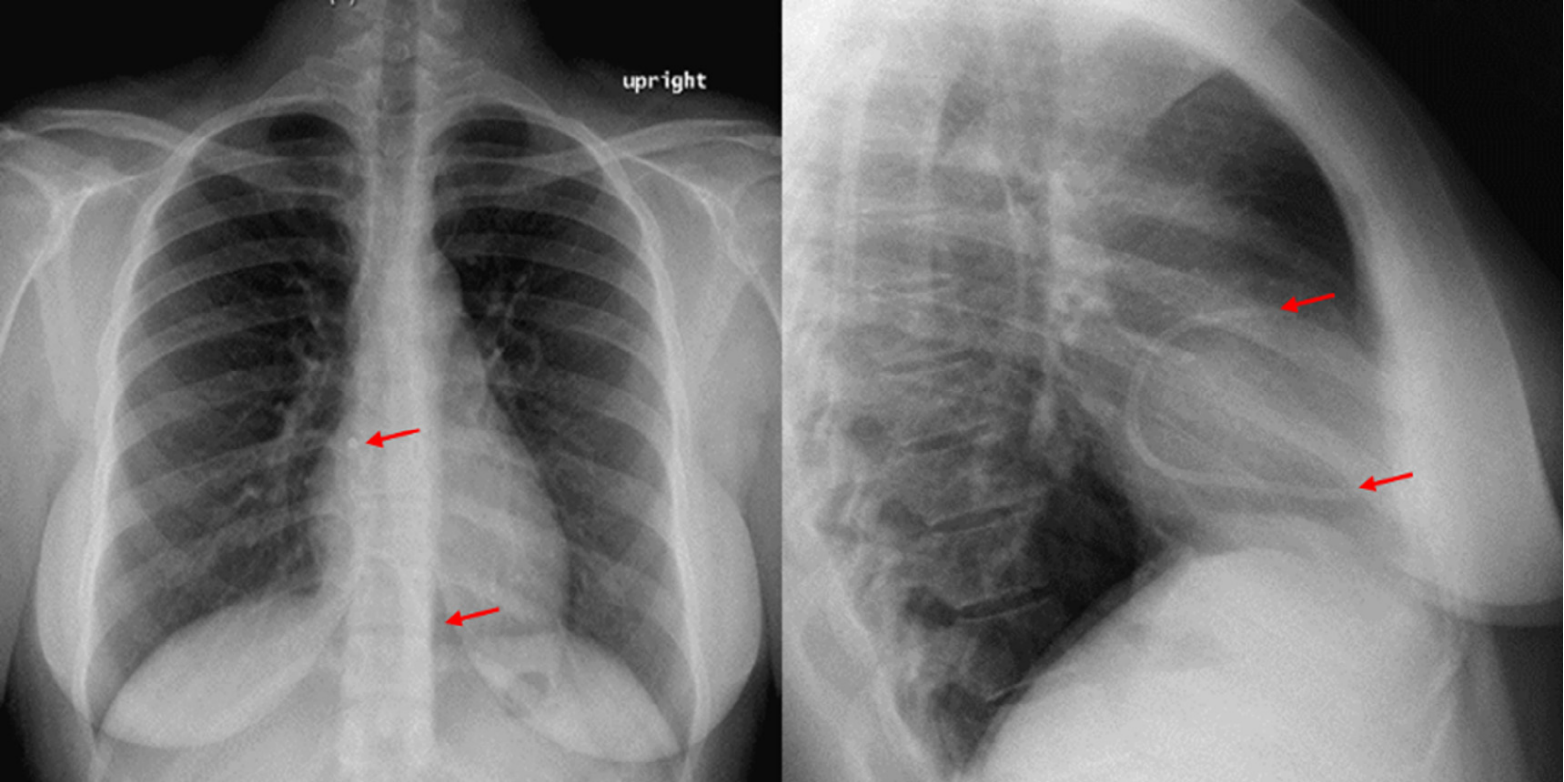
Chest X‐ray in PA (left) and lateral (right) views demonstrating the presence of a large retained narrowed catheter/tubing within the right atrioventricular region measuring approximately 10 cm in overall length. Red arrows identify fragment ends.

Initial examination revealed stable vital signs and normal cardiac, chest, pulmonary, and abdominal examination. Complete blood count and basic metabolic panel were unremarkable. ECG showed normal sinus rhythm. Lateral and PA chest X‐rays (Fig. 1) revealed a 10‐cm narrow foreign body within the right atrioventricular region consistent with a retained catheter fragment. The patient had no prior X‐rays for comparison. Bedside transthoracic echocardiography revealed no gross abnormalities of the right ventricle or tricuspid valve with a large, fixed foreign body noted in the right atrium without thrombus, contractile changes, or effusions. What percentage of indwelling tunneled catheters fracture, and how should this retained fragment be removed?

More than five million central venous catheters are placed annually within the United States 1. Catheter fracture occurs in approximately 2–3% of patients 2, 3, and unidentified fragments are presumably rare. This patient underwent successful fluoroscopy‐guided removal of the fragment from the right atrium without complication and in a single piece. This was achieved with use of a 7‐French snared catheter introduced through a 10‐French sheath via the right common femoral vein. The fragment was found to be a single lumen, portacath‐type device >11 cm in length. After removal, the patient was completely relieved of symptoms, which possibly can be attributed to mechanical irritation of cardiac afferents. Despite this success and others 4, there are reported failures 5, and there is no clear consensus on optimal management and removal of fractured catheters despite the significant risks posed.

## Authorship

CM: drafted the article. UT and MG: provided documentation of the patient's clinical course. CM, UT, and MG: contributed to review and revision. CM, UT, and MG: approved the final article and are accountable for all of its aspects.

## Conflict of Interest

None declared.
